# Genome-nanosurface interaction of titania nanotube arrays: evaluation of telomere, telomerase and NF-κB activities on an epithelial cell model

**DOI:** 10.1039/d1ra05325f

**Published:** 2022-01-14

**Authors:** Rabiatul Basria S. M. N. Mydin, Srimala Sreekantan, Darius Widera, Khairul Arifah Saharudin, Roshasnorlyza Hazan, Mustafa Fadzil Farid Wajidi

**Affiliations:** Oncological and Radiological Sciences Cluster, Advanced Medical and Dental Institute, Universiti Sains Malaysia 13200 Bertam, Kepala Batas Pulau Pinang Malaysia rabiatulbasria@usm.my +60-04-5622351; School of Materials and Mineral Resources Engineering, Universiti Sains Malaysia Engineering Campus, 14300 Nibong Tebal, Seberang Perai Selatan Pulau Pinang Malaysia; Reading School of Pharmacy Whiteknights Reading UK RG6 6U; Qdos Interconnect Sdn Bhd No 99 Bayan Lepas Industrial Estate 11900 Penang Malaysia; Materials Technology Group, Industrial Technology Division, Nuclear Malaysia Agency Bangi, Kajang 43000 Selangor Malaysia; School of Distance Education, Universiti Sains Malaysia 11800 Penang Malaysia

## Abstract

Titanium dioxide nanotube arrays (TNAs) provide a promising platform for medical implants and nanomedicine applications. The present cell–TNA study has provided profound understanding on protection of genome integrity *via* telomere, telomerase and NF-κB activities using an epithelial cell model. It has been revealed in this study that cell–TNA interaction triggers the telomere shortening activity and inhibition of telomerase activity at the mRNA and protein level. The present work supported that the cell–TNA stimulus might involve controlled transcription and proliferative activities *via* NBN and TERF21P mechanisms. Moreover, inhibition of NF-κB may promote molecular sensitivity *via* senescence-associated secretory phenotype activities and might result in reduced inflammatory response which would be good for cell and nanosurface adaptation activities. Thus, this nanomaterial-molecular knowledge is beneficial for further nanomaterial characterization and advanced medical application.

## Abstract 2

Schematic illustrations described the activation of pro-survival and cellular senescence activities *via* NF-κb inhibition upon the interaction from cells grown on TNA nanosurfaces.

## Graphical abstract

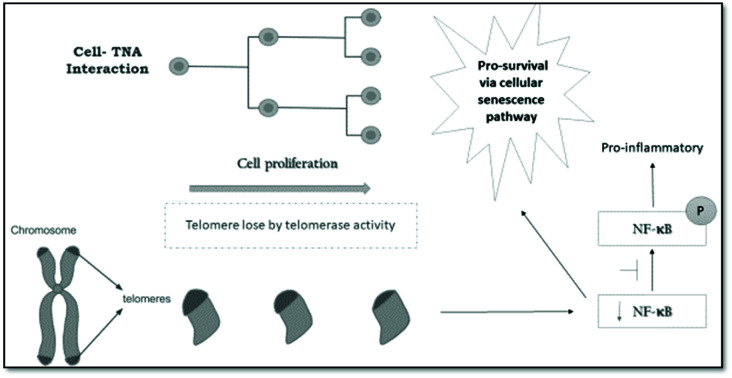

## Introduction

Titanium dioxide nanotube arrays (TNAs) have been widely studied as a nanosurface for biomedical applications especially in biosensors, drug delivery, diagnostics, and dental and orthopedic implants.^1^ The nanometric scaled topography of biomedical products plays a decisive role in the surrounding tissue acceptance, cellular stability and cell survival.^2^ The fate of any cell is determined by its adaptive capacity to an environment.^3^ The mislaying of decisions related to the cell's fate may result in cellular transformation or carcinogenesis risk.^4^

Therefore, studying the nanomaterial potential interaction at the molecular level of the cells including proteins and DNA may allow further understanding on nanomaterial and genome integrity. Bio-indicators for genome integrity such as telomere and telomerase crucial for both cancer and aging *via* cellular senescence mechanisms.^5–11^ Cellular senescence described by the steady and long-lasting proliferative inability, in spite of prolonged viability and metabolic activity.^12^

Cells undergoing senescence associated either from telomere dysfunction, DNA damage, or alterations in oncogenes that exhibiting intense transcriptomes variations. These mechanisms relayed on nuclear factor-kappa B (NF-kB) transcription factors which are also linked to an inflammatory transcriptome activation thus reinforcing the senescent cell-cycle arrest. The growth arrest *via* senescence is permanent and is triggered by multiple stress stimuli such as stress from cellular adaptation process.^13–17^

Studying the senescent pathway in this work provides further understanding on the potential of cell-nanomaterial molecular interactions in determining the cell's fate. Epithelial cell line is one of the cell lines model studied for biomaterial-molecular characterization. Therefore, present work studied the cell–TNA interaction on transcription controls *via* NF-κb, telomere and telomerase on epithelial cell lines model.

## Experimental section

### Study materials

In this experiment, HT-29 cells grown on the TNA, bare titanium foil (TiP) and glass surface (GS-Thermo Scientific Nunc) were studied. The dimensions of each test materials were standardized at 2 cm × 2 cm (*L* × *W*). TNA were prepared according to the protocol earlier^17^ with properties as follows: 100 nm outer diameter, 60 nm inner diameter, 15 nm wall thickness and 300 nm length.

### Cell culture

Epithelial cell lines model, HT-29 from the American Type Culture Collection (USA) were cultured following protocol described previously.^18^ Cells grown on each study material surfaces such were studied. Additionally, cells grown on culture system surface (plastic) has also been studied which represent as negative control. Whereas, plastic surface treated nicotine (NQ) represented as positive control for oxidative stress and oxygen tension environment that eventually inhibits the cell growth, proliferation and differentiation activities.^19^ Each test materials were seeded with cells at density of 1 × 10^5^ cell per well until the cells reach 90–95% confluence.

### Telomere length assessment

Metaphase/interphase slides for qualitative telomere length assessment by fluorescence *in situ* hybridization (FISH) using peptide nucleic acid (PNA) probes. Centromere probe were used as standard. Further hybridization methods were conducted as described according to manufacturer's instruction for Panagene kit (Daejon, South Korea). The centromere conjugated with cyanine orange-fluorescent dye (cy3) and telomere conjugated with green fluorescein isothiocyanate (FITC) were used for detection respectively. The slides were mounted and examined using phase-contrast microscope (Olympus BX51, Japan). For each slide, 60 good quality metaphases were visualized under oil immersion (1000×) for total chromosomes profiles (total chromosomes number, dicentric (Dic), acentric (Ace) ring chromosomes). At least 10% of 100 cells analysed in ten different fields, were scored as positive for FISH signals^19^ gene amplification with statistical analysis (Student's *t*-test).

Quantitative telomere length assessment by terminal restriction fragment (TRF) length were performed according to TeloTAGGG Telomere Length Assay Kit (Roche Diagnostics). DNA extractions were extracted according to manufacturer's instruction for GeneJETTM Genomic DNA Purification Kit (Fermentas). Two μg of extracted genomic DNA for each sample was electrophoresed in a 1% (w/v) Tris-acetate agarose gel at 70 V for 90 minutes and subjected to terminal restriction fragment (TRF) assessment by Southern blotting technique. TRF length of each sample was determined using the TeloTAGGG Telomere Length Assay Kit given by manufacturer's instructions (Roche Diagnostics) using chemiluminescent signal and VersaDoc software (BioRad). The mean length for TRF was estimated by comparing each fragment with a known molecular weight standard from Roche Kit. Student's *t-*test was used for the statistical analysis while the TRF was calculated using the following formula:

1 TRF = Σ(ODi)/Σ(ODi/Li)

where ODi is the chemiluminescent signal and ODi/Li is the length of the TRF position.

### Telomerase expression analyses

Qualitative and quantitative telomerase activity was measured by telomeric repeat amplification protocol (TRAP) using the TeloTAGGG Telomerase PCR Elisa PLUS kit (Roche Diagnostics) based on the manufacturer's instructions. The telomeric repeat amplification products were then visualized by a polyacrylamide gel electrophoresis (PAGE) technique. The products were mixed with 5 μL of 5× sample buffer (BioRad) dye and separated using 12.5% PAGE for 90 minutes at constant current of 120 V using Bio-Rad electrophoresis systems. After electrophoresis, the separated PCR products on the gel were transferred by Southern blotting onto a positively charged membrane. The membrane was then washed twice with sterile distilled autoclaved water and treated with 2% (v/v) blocking buffer for 30 minutes. Later the membrane was hybridized with a streptavidin-alkaline phosphatase (AP) conjugate for 1 hour. The chemiluminescence techniques were used to visualize the blotted products.

The detection procedure was performed according to manufacturer's instruction from Roche kit. The PCR amplification products were denatured and hybridized with digoxigenin-labeled detection probes. The simultaneously amplified internal standard (216 bp) present in the reaction mixture was used to exclude false negative results due to Taq DNA-polymerase inhibitors that were eventually present in the cell lysates. The resulting products were next immobilized in the well of a streptavidin-coated 96-well microplate and hybridized with a digoxigenin (DIG)-labelled probe specific (telomeric repeat sequence). Immobilized amplicons were then detected with an antibody against digoxigenin, which was conjugated to horseradish peroxidase (HRP) and then quantified photometrically at an absorbance of 450 nm against the blank value (reference wavelength of 690 nm). All samples were tested in duplicate, and the result for a sample is the average of the triplicate sample values. A control template with a known amount of high and low telomerase activity was used to determine the relative telomerase activity (RTA).

### mRNA expression profile by real time PCR

The genes of interest were selected based on the pathway that are involved telomere extension by telomerase, telomerase signalling and NF-κB activities. The genes information was obtained from the website of National Centre of Biotechnology Information (NCBI) database. The validated gene sequence primers were selected from TaqMan Assay by Life Technologies, Applied Biosystems (2012) based on the reference sequence number and correct assay ID which contained three set of one manufacturing control (18S rRNA), housekeeping genes (EGFR and HNRNPA2B1) and genes of interest (Table 1).

**Table 1:** The list of target genes reference sequence and assay ID for real-time PCR analysis

No.	Genes symbol	Ref. Seq.	Assay ID
1	18S: ribosomal RNA	X03205	HS99999901_s1
2	EGFR: epidermal growth factor receptor	NM_201282.1	Hs01076078_m1
3	HNRNPA2B1: heterogeneous nuclear ribonucleoprotein A2/B1	NM_031243.2	Hs00242600_m1
4	TERF1: telomeric repeat-binding factor 1	NM_017489.2	Hs00819517_mH
5	TERF2: telomeric repeat-binding factor 2	NM_005652.3	Hs00194619_m1
6	POT1: protection of telomeres 1	NM_015450.2	Hs00209984_m1
7	NBN: cell cycle regulatory protein-P95	NM_002485.4	Hs01039836_m1
8	TERF2IP: telomeric repeat binding factor 2-interacting protein	NM_018975.3	Hs00430292_m1
9	TINF2: TERF1 (TRF1)-interacting nuclear factor 2	NM_012461.2	Hs00173291_m1
10	TERT: telomerase reverse transcriptase	NM_198253.2	Hs00972656_m1
11	TERC: telomerase RNA component	NR_001566.1	Hs03454202_s1
12	TNKS: tankyrase, TRF1-interacting ankyrin-related ADP-ribose polymerase	NM_003747.2	Hs00186671_m1
13	TEP1: telomerase-associated protein 1	NM_007110.4	Hs00200091_m1

The protocol from RNeasy mini kit (Qiagen) and High Capacity RNA-to-CDNA Kit (Applied Biosystems) were used to for study sample preparation. The real-time polymerase chain reaction (RT-PCR) assays were performed using TaqMan® Array 96-Well Fast Plates and thermal cycler StepOnePlus systems at thermal profile of 95 °C 20 s/[95 °C 3 s − 60 °C 30 s] × 40. The data obtained from the gene expression assay were analyzed by comparative cycle threshold method and relative expression of the housekeeping genes. Further statistical analysis was performed using one-way analysis of variance (ANOVA) with the help of SPSS software version 22.

### Protein analyses

The protein extraction was performed using extraction buffer from Invitrogen (FNN0011). Each sample with 20 μg concentration were separated using 10% sodium dodecyl sulfate (SDS) polyacrylamide gels and transferred at 200 mA (2 hours) electrophoretically to a polyvinylidene difluoride (PVDF) membrane (0.45 μm pore size, Millipore).

Protein samples were collected from cells cultivated on the surface of TNA (N), Ti (TiP), glass (G), plastic (P) and plastic + nicotine (NQ). The expression on the surface N was compared to that of the surface T. Meanwhile, the protein expressions were also compared to surface NQ which represented the negative control for cell growth inhibited by the presence of oxidative cellular stress as described by Argentin and Cicchetti (2004).^20^

The detection of targeted protein expression was performed according to standard protocol described in SMN Mydin, R. B., *et al.* (2017).^18^ Antibodies used were from the following: human telomerase reverse transcriptase (hTERT) Rabbit Monoclonal (Millipore, #MABE14) with dilution factor 1 : 1000, Goat Anti-Rabbit IgG (H+L) HRP (Cell Signalling) with dilution factor 1 : 3000, nuclear factor kappa light chain enhancer of activated B cells (NF-kB-p65) Mouse Monoclonal (Invitrogen, Lot #982026D) with dilution factor 1 : 1000, Anti-Mouse IgG (Fc specific)-Peroxidase (Sigma,#A0168), Anti-rabbit IgG, HRP-linked antibody (Cell Signaling, 7074) with dilution factor 1 : 5000 and Beta-Actin Polyclonal Rabbit (Cell Signalling, 4967S) with dilution factor 1 : 1000 was used as a reference signal. The experiment was done for two biological samples. The values for each of the protein expressions were further normalized with reference signal. Further analysis on fold-change of the targeted protein expression was calculated by relative and normalized data.

## Results

### Telomere length profile

**Fig. 1:**
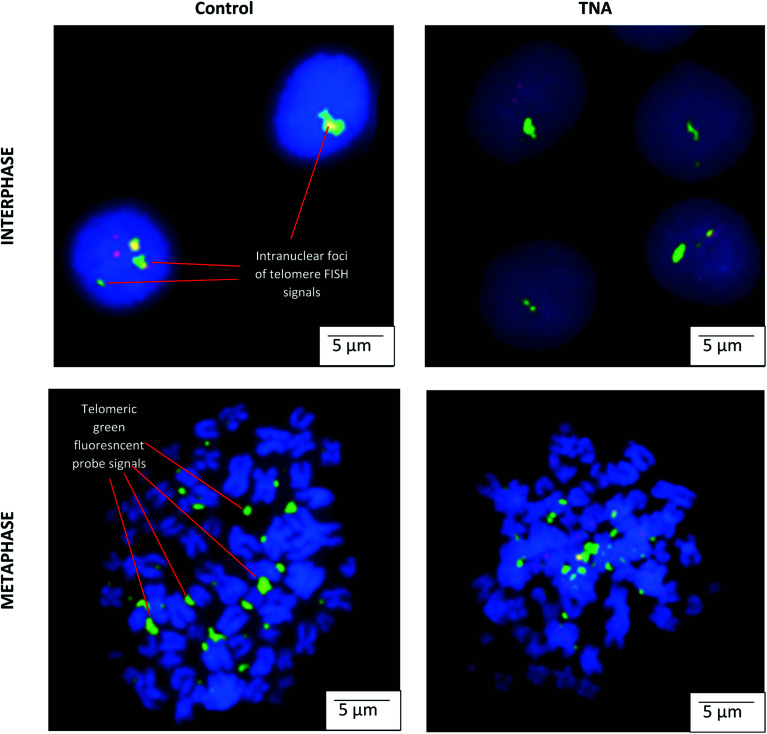
Localization of telomere region detected by fluorescence *in situ* hybridization probe signals. Representative images show the epithelial HT-29 cells cultivated on TNA and control (plastic surfaces). Telomeric DNA signals (FITC, green) were observed in interphase and metaphase cells. The chromosomes were counterstained with DAPI (blue), original magnification of 1000×. Note the decreased telomeric signals observed on the cell cultivated on TNA surface. Bar = 5 μm.

**Fig. 2:**
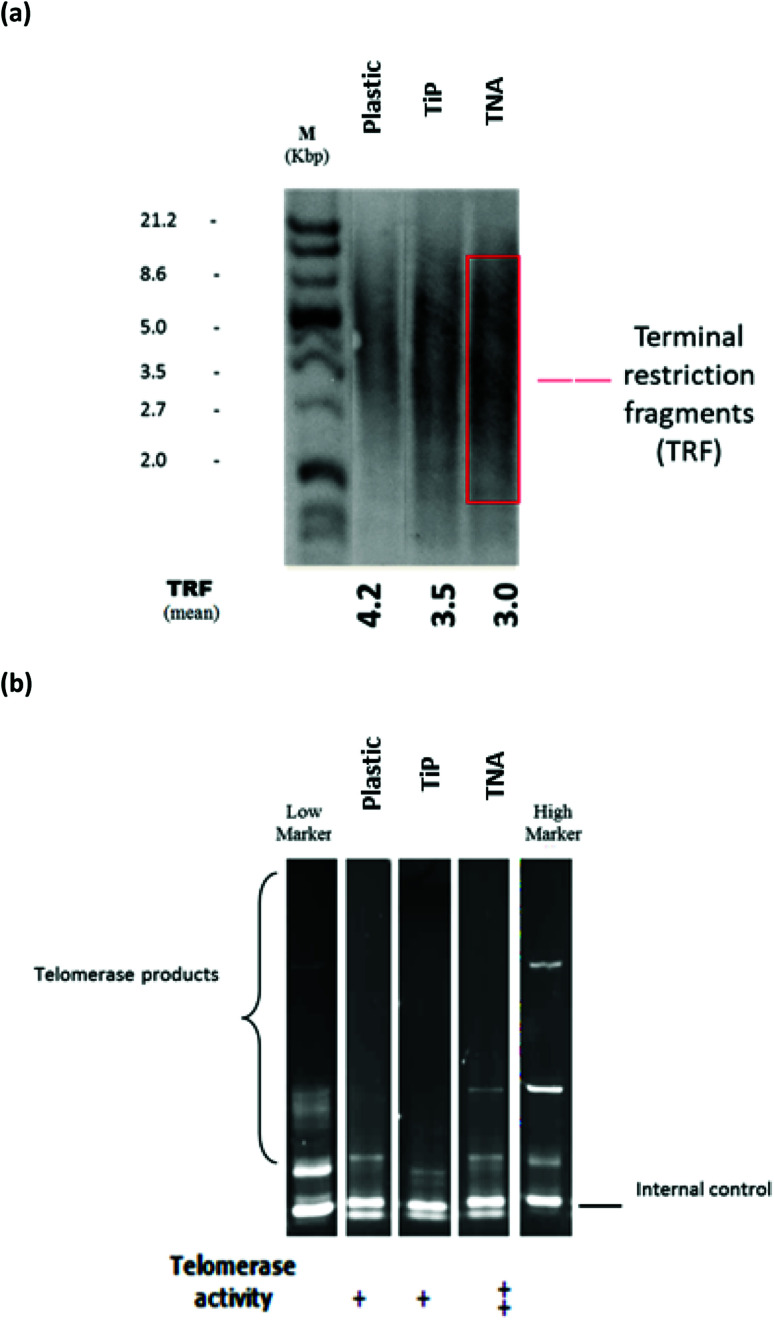
Telomerase activity was observed on epithelial-HT29 cells cultivated with the TNA at 7 days incubation periods. Epithelial HT-29 cells were cultured on P, TiP and TNA surfaces. (a) Telomerase activity was determined *via* Southern blot and chemiluminescence detection of the PCR amplified telomerase products. (b) Graphic presentation of mean telomerase specific signal intensity that was quantified photometrically using ELISA detection protocol. Cells grown on TNA exhibited higher telomerase expression increase compared to other surfaces.

The microscopic examination on interphase and metaphase cells hybridized with a telomere-specific fluorescent PNA probe (FISH) revealed the presence of telomere length for each of the samples. In the control counterpart, distinctively large and very bright intra-nuclear foci of telomere FISH signals were observed (Fig. 1). The analysis indicated the presence of decreased telomeric signals in cells cultivated on TNA surface (20% positive cells) in comparison with reference (40% positive cells). The strength of fluorescence intensity on the telomeric signals was proportionately related with the length of telomeric DNA. Thus, cells cultivated on TNA surface may exhibit telomere shortening. This finding was also in agreement with quantitative telomere assessment *via* Southern blotting technique observed in Fig. 2a. TRF value of cells grown on TNA (3.0 Kbp) was lower compared to cells grown on control surfaces such as P (4.2 Kbp), G (4.0 Kbp) and TiP (3.5 Kbp). Data were obtained from two independent test.

### Telomerase expression profile

The telomerase activities were profiled by telomeric repeat amplification protocol (TRAP) gel and ELISA detections (Roche). Telomerase activities on cells cultivated on different material surfaces were compared with high and low markers (obtained together in the Roche kit). Telomerase activity of epithelial HT-29 cells grown on the TNA was found to be relatively higher by 1.2-fold compared to that of P and 1.4-fold increase compared to that of TiP, as shown in Fig. 2b. Similar patterns were observed from (TRAP) gel and ELISA detections (Roche).

### Expression of genes involved in telomere regulation mechanism

**Fig. 3:**
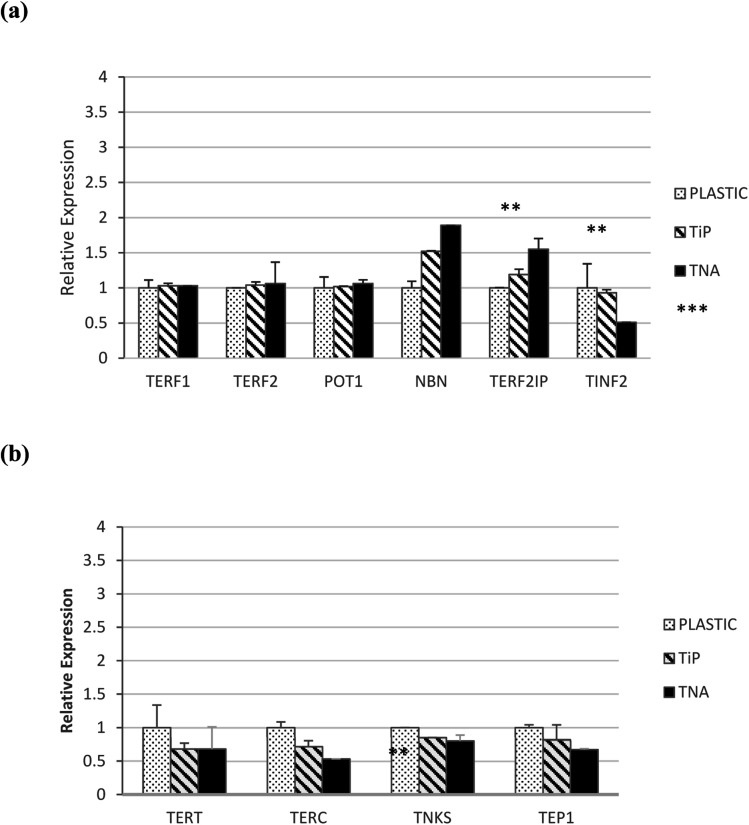
Expression of genes involved in (a) telomere regulation mechanism and (b) telomerase modulation mechanism on epithelial HT-29 cells model. The study material surfaces were P, TiP and TNA. Data points represent means ± SEM of triplicate observations from a representative experiment *p < 0.05 (significant), **p < 0.01 (very significant) and ***p < 0.001 (extremely significant) based on one-way ANOVA.

Telomere regulation mechanism were studied on epithelial HT-29 cells grown with different material surfaces involving TERF1, TERP2, POT1, NBN, TER21P and TINF2 genes (Fig. 3a). NBN (*p* < 0.001) and TER21P (*p* < 0.01) genes showed significant expressions increased pattern on TNA surface compared to their pattern on other surfaces. Meanwhile, TINF2 expression showed decreasing pattern on TNA compared to its pattern on other surfaces. Lastly, the expression of TERF1, TERF2 and POT1 did not show any significant changes. These findings suggested that cell–TNA interaction may involve telomere regulation mechanism.

### Expression of genes involved in telomerase modulation mechanism

Epithelial HT-29 cells also showed a decreased pattern of TERT expression on TNA surface, however the value did not appear to be sufficiently significant (*p* > 0.05). The genes involved in telomerase modulation were further profiled on TEP1, TERC and TNKS genes (Fig. 3b). Decreased expression patterns of TEP1, TERC and TNKS genes for TNA surface compared to its expression on other surfaces in epithelial HT-29 cells was observed. These findings suggested that cell–TNA interaction may involve telomerase modulation mechanism possibly by TERC activity.

### hTERT and NF-κB proteins analyses

**Fig. 4:**
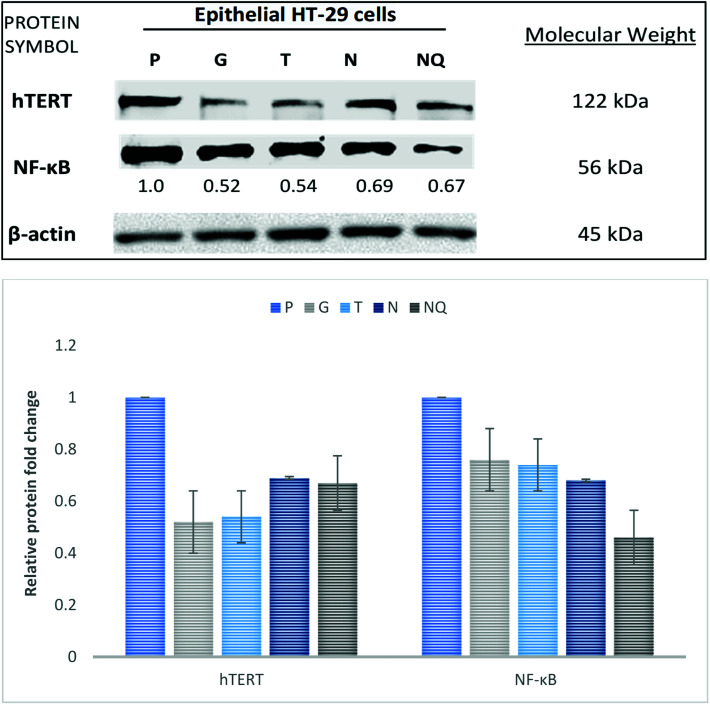
Immunoblot analysis on hTERT and NF-κb protein expression. The studied protein samples were obtained from TNA (N), TiP (T), glass (G), plastic (P) and plastic + nicotine (NQ). Differential protein expressions of hTERT and NF-κB were expressed in epithelial HT-29 on the study materials. β-Actin protein profile was used as a loading control. Results were obtained from two biological repeats.

The involvement of cell–TNA interaction in DNA damage and cell cycle check point was studied at hTERT and NF-κB protein levels using epithelial HT-29 on different material surfaces. The protein expression of hTERT was detected using immunoblotting at 122 kDa molecular weight (Fig. 4). The epithelial HT-29 cells grown on surface N showed decreased hTERT protein expression at 0.69-fold change in comparison with its expression on surface P at 1-fold change. hTERT expression was slightly increased on surface N compared to the cells on surface G (0.52-fold change), surface T (0.54-fold change) and control surface with NQ treatment (0.67-fold change). Next, the expression of NF-κB protein was detected at 56 kDa molecular weight. The epithelial HT-29 cells grown on surface N showed decreased NF-κB expression at 0.68-fold in comparison with its expression on surface P (1-fold change), surface G (0.76-fold change) and surface T (0.74-fold change). The cells on control surface with NQ treatment showed much decreased NF-κB expression at 0.46-fold change. Therefore, cell–TNA interaction showed inhibition of cell cycle check point proteins such as hTERT and NF-κB proteins.

## Discussion

Findings from this study observed cellular stimulus from cell–TNA interaction resulted in telomere shortening (Fig. 1 and 2a). The telomere integrity may be regulated by several genes such as telomeric repeat-binding factor 1 (TERF1), telomeric repeat-binding factor 2 (TERF2), protection of telomeres 1 (POT1), cell cycle regulatory protein-P95 (NBN), telomeric repeat binding factor 2-interacting protein (TERF2IP) and TERF1 (TRF1)-interacting nuclear factor 2 (TINF2).^21^ In this study, the possible cell–TNA interactions were predicted by significant up-regulation of the NBN and TERF2IP genes in epithelial HT-29 cells. NBN expression is crucial in protecting the telomere integrity from DNA damage-induced checkpoint and the expression of TERF2IP gene may involve in regulating telomere length and protector as the shelterin complex (telosome).^21,22^ Noted that the telomere region is more hypersensitive to DNA damage activity under cellular stress condition^22–24^ which may be resulted from the initial cellular adaptation response on TNA nano-surface.

The mechanism of telomere regulation occurs with the presence of telomerase activity or the alternative elongation of telomeres (ALT) pathway.^25^ It was observed in the present study that epithelial HT-29 cells may possess indefinite proliferative activity cells with the presence of telomerase activity in order to maintain the telomere function (Fig. 2b). Note that, a continuous and uncontrolled proliferative activity would lead to genomic disarray and carcinogenesis risk.^26^ Therefore, the mechanism of senescence would be important in maintaining a secure withdrawal from the proliferation occupied by the tumour suppressor mechanism. In this study, the telomere loss would limit cell proliferation *via* controlled proliferative activities as discussed earlier.^27^ This mechanism will not directly involve the apoptosis pathway, but would lock the cell in the G1-phase of the cycle.^27,28^ Besides, the programming of the cells for senescence, during the process of cell cycle arrest may allow the cells to activate several genes and protein cellular adaptation towards TNA nanostructure in order to survive.

Conventionally, the shortening activity of telomeres is accompanied by the suppression of telomerase reverse transcriptase (TERT) expression, arguably claimed to have contributed to an anticancer mechanism.^29^ The findings in this study have shown consistent suppression of TERT activity at the mRNA (Fig. 3b) and protein level (Fig. 4) on cells cultivated with TNA nanosurface in epithelial HT-29. Moreover, the suppression of telomerase-associated protein 1 (TEP1) and telomerase RNA component (TERC) expression at mRNA level was also observed in this study on the cells cultivated with TNA nanosurface (Fig. 3b), which supported reduced telomerase activity.^30^ Hence, the findings from this study have suggested that cell–TNA stimulus would result in repression of TERT activity and contribute to the telomere shortening and senescence mechanism.

Besides, it has been debated that TERT also has another function at the post-transcription level, which is independent of its telomerase activity.^31^ Telomerase enzyme activity by TRAP assay was increased on cells cultivated with TNA nanosurface in epithelial HT-29 cells (Fig. 2b). This activity may contribute to the regulation of gene expression and interact with transcription factors (*e.g.*, p65, β-catenin or BRG1), thus may control the gene transcription programmes *via* NF-κB signalling and the Wnt/β-catenin pathway. NF-kB signalling promotes IKK-mediated phosphorylation of RELA/p65, activating expression genes involved in a targeted cellular transcription activity.^32,33^ The findings in this study have indicated that the expression of TERF2IP (Fig. 3b) may interact with NF-κB signalling pathway and impact as well as regulate the transcription activity of the cells that are cultivated on TNA nanosurface. Moreover, NF-κB signalling also be responsible for the mitochondrial function and DNA/ROS (reactive oxygen species) damage responses. Furthermore, the tankyrase, TRF1-interacting ankyrin-related ADP-ribose polymerase (TNKS) expressions of mRNA in this study did not present any significant changes (Fig. 3b) between the cells grown on TNA and other material surfaces. It can be suggested that the expression of these genes may support the Wnt signalling pathway *via* the signal transduction pathways and vesicle trafficking mechanism. The molecular mechanism of these genes may assist the cellular homoeostasis and the adaptation process towards the nanosurface structure of TNA.

This study has provided insights into the expression of hTERT by NF-κB pathway. Note that, the nuclear factor κB (NF-κB) controls the gene expression responsible in pro-survival and pro-inflammatory activities.^34^ As a result of this study, it is suggested that cell–TNA interaction would activate the pro-survival activities *via* cellular senescence pathway. The inhibition of NF-kB and TERT expression was found as a protein level (Fig. 4). A study by Wang and Bennett (2010)^28^ has reported that inhibition of NF-kB can reduce the TERT activity or overexpression. Moreover, inhibition of NF-κB may promote molecular sensitivity *via* cellular senescence and interaction to cytokines, which is referred to as “senescence-associated secretory phenotype” (SASP).^35^

**Fig. 5:**
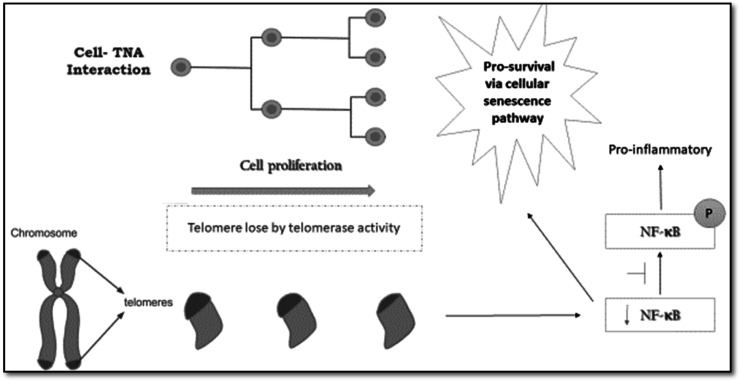
Schematic illustrations described the activation of pro-survival and cellular senescence activities *via* NF-κb inhibition upon the interaction from cell grown on TNA nanosurface. This mechanism was predicted based on the epithelial HT-29 cell line model.

On the other hand, these results also suggested cell–TNA interaction might involve in the pro-inflammatory response as described in Fig. 5. Telomerase and NF-kB expression have been reported as the master regulator in inflammation and cancer progression. The telomerase mechanism would directly regulate NF-κB-dependent gene expression by binding to the subunit of p65 (NF-κB) and subsequently recruit to NF-κB promoters' subset such as those of IL-6 and TNF-α and cytokines.^36^ The hypoxia condition that pumps energy to facilitate the cell movement and adaptation on TNA nano-surface structure may lead to cellular stress. This interaction may trigger the inflammatory response to counterbalance the presence of oxidative stress.^37,38^ However, the severe inflammatory response might interrupt the cellular adaptation activities.^39,40^ Therefore, suppression of NF-kB protein observed in the cell–TNA interaction may be associated with reduced inflammatory response, which is good for future cellular adaptation feedback.

## Conclusions

The cell fate decision on cell–TNA interaction have showed could regulates telomere shortening, hTERT and NF-κB signaling pathway which involved in the protection of genome integrity *via* cellular senescence. This event is crucial in pro-survival and pro-inflammatory activities as adaptive response to the nanostructure environment. The knowledge from this study will help to refine the TNA properties for better future prospect in biomedical applications.

## Author contributions

All authors contributed equally. The manuscript was written through contributions of all authors. All authors have given approval to the final version of the manuscript.

## Conflicts of interest

The authors declare that they have no competing interests.

## Supplementary Material
